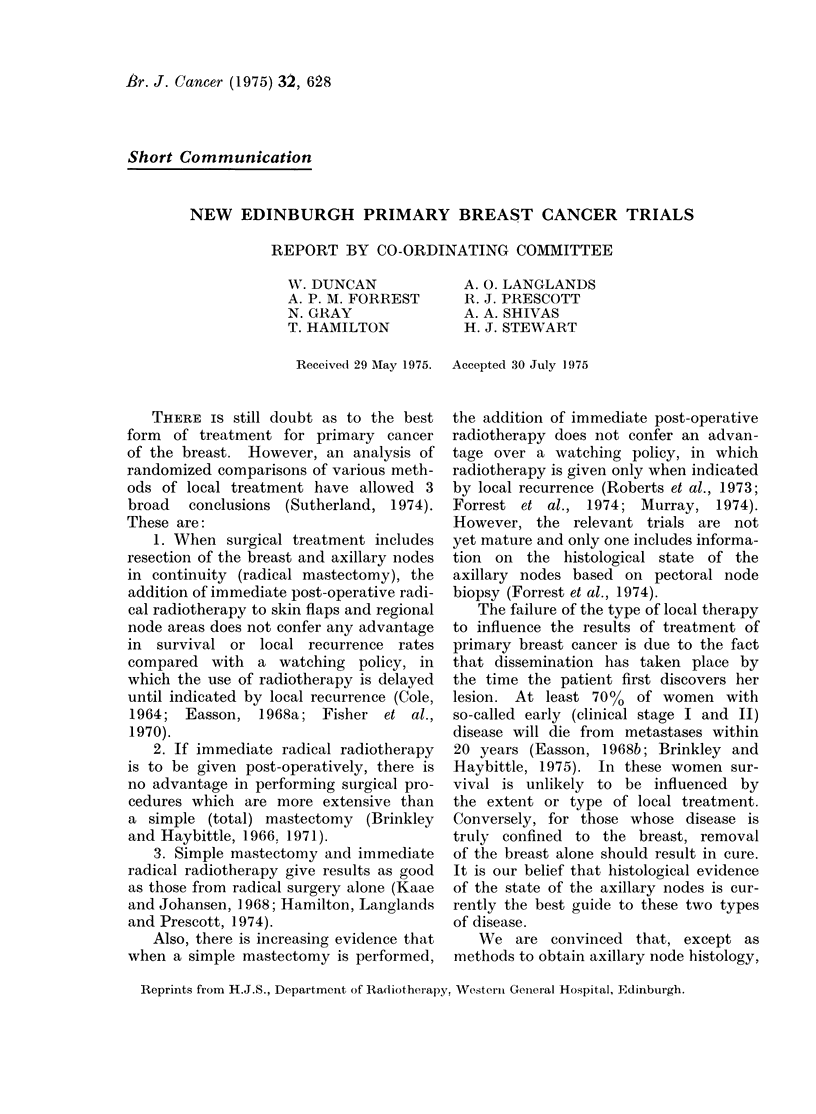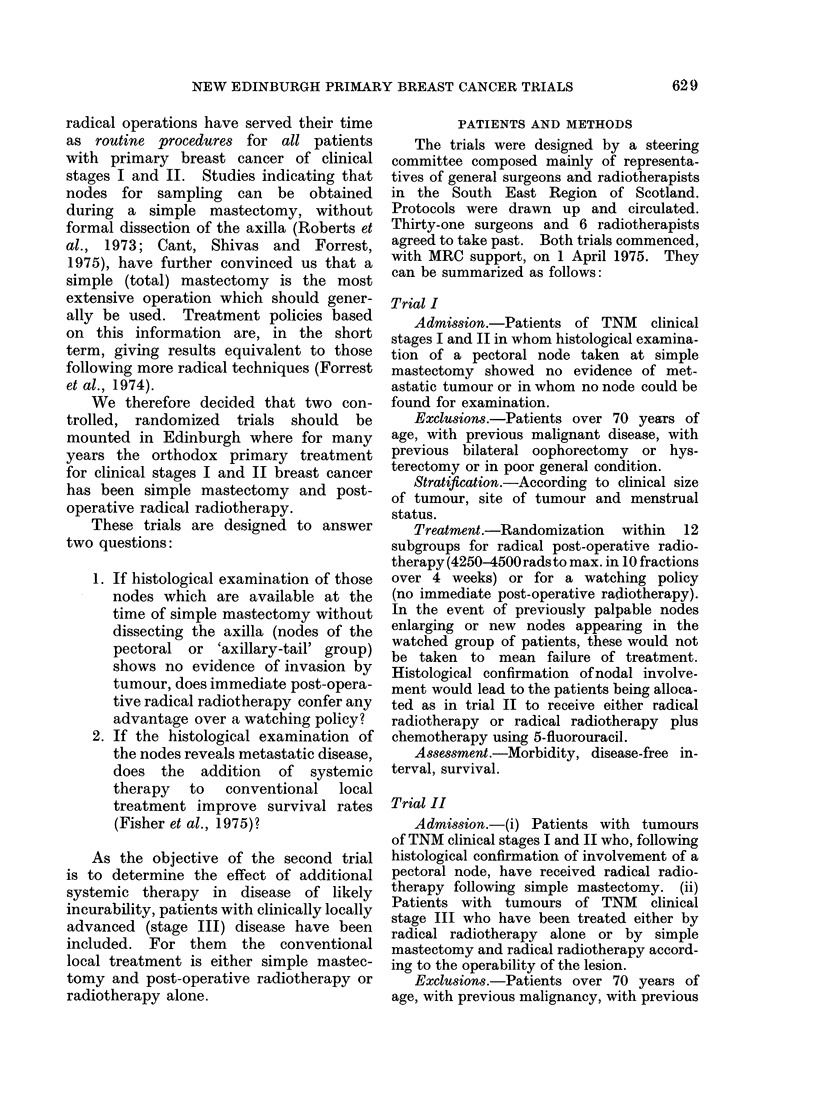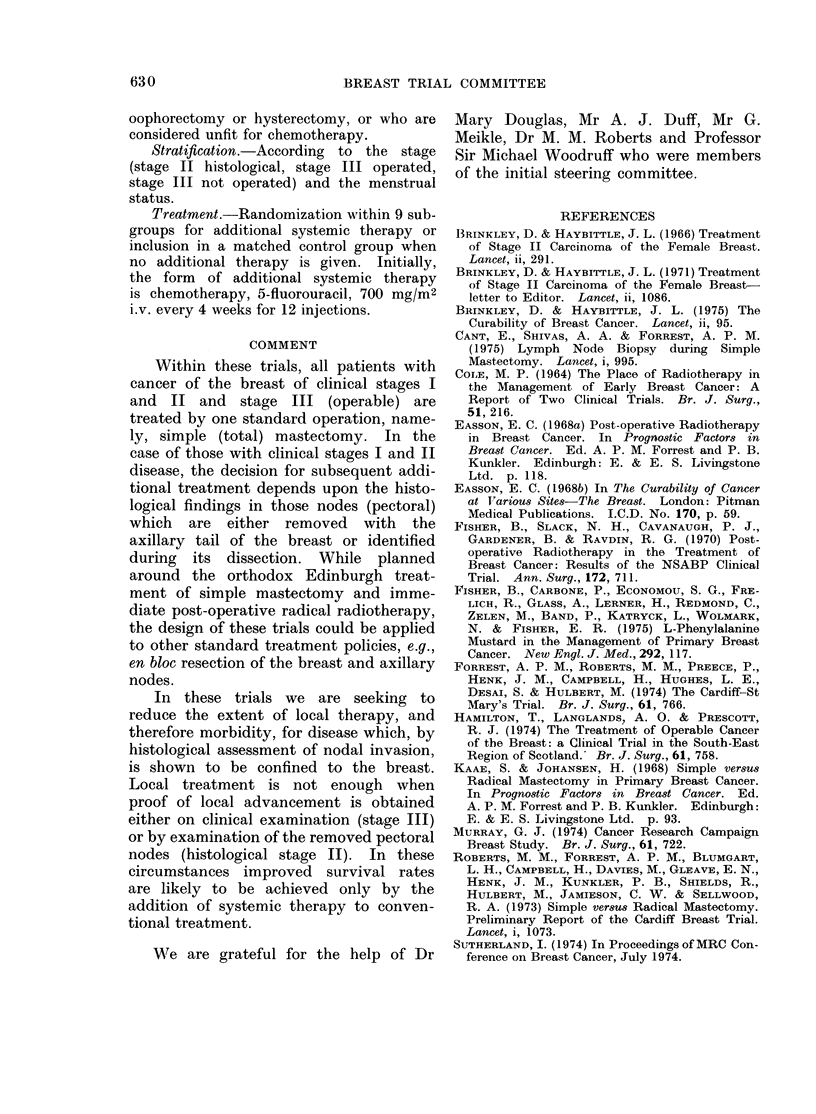# New Edinburgh primary breast cancer trials.

**DOI:** 10.1038/bjc.1975.270

**Published:** 1975-11

**Authors:** 


Br. J. Cancer (1975) 32, 628

### Short Communication

### NEW EDINBURGH PRIMARY BREAST CANCER TRIALS

REPORT BY CO-ORDINATING COMMITTEE

W. DUNCAN

A. P. M. FORREST

N. GRAY

T. HAMILTON

A. O. LANGLANDS

R. J. PRESCOTT

A. A. SHIVAS

H. J. STEWART

Received 29 May 1975. Accepted 30 July 1975

THERE IS still doubt as to the best form of treatment for primary cancer of the breast. However, an analysis of randomized comparisons of various methods of local treatment have allowed 3 broad conclusions (Sutherland, 1974). These are:

1. When surgical treatment includes resection of the breast and axillary nodes in continuity (radical mastectomy), the addition of immediate post-operative radical radiotherapy to skin flaps and regional node areas does not confer any advantage in survival or local recurrence rates compared with a watching policy, in which the use of radiotherapy is delayed until indicated by local recurrence (Cole, 1964; Easson, 1968a; Fisher *et al.*, 1970).

2. If immediate radical radiotherapy is to be given post-operatively, there is no advantage in performing surgical procedures which are more extensive than a simple (total) mastectomy (Brinkley and Haybittle, 1966, 1971).

3. Simple mastectomy and immediate radical radiotherapy give results as good as those from radical surgery alone (Kaae and Johansen, 1968; Hamilton, Langlands and Prescott, 1974).

Also, there is increasing evidence that when a simple mastectomy is performed, the addition of immediate post-operative radiotherapy does not confer an advantage over a watching policy, in which radiotherapy is given only when indicated by local recurrence (Roberts *et al.*, 1973; Forrest *et al.*, 1974; Murray, 1974). However, the relevant trials are not yet mature and only one includes information on the histological state of the axillary nodes based on pectoral node biopsy (Forrest *et al.*, 1974).

The failure of the type of local therapy to influence the results of treatment of primary breast cancer is due to the fact that dissemination has taken place by the time the patient first discovers her lesion. At least 70% of women with so-called early (clinical stage I and II) disease will die from metastases within 20 years (Easson, 1968*b*; Brinkley and Haybittle, 1975). In these women survival is unlikely to be influenced by the extent or type of local treatment. Conversely, for those whose disease is truly confined to the breast, removal of the breast alone should result in cure. It is our belief that histological evidence of the state of the axillary nodes is currently the best guide to these two types of disease.

We are convinced that, except as methods to obtain axillary node histology,

Reprints from H.J.S., Department of Radiotherapy, Western General Hospital, Edinburgh.

radical operations have served their time as *routine procedures* for *all* patients with primary breast cancer of clinical stages I and II. Studies indicating that nodes for sampling can be obtained during a simple mastectomy, without formal dissection of the axilla (Roberts *et al.*, 1973; Cant, Shivas and Forrest, 1975), have further convinced us that a simple (total) mastectomy is the most extensive operation which should generally be used. Treatment policies based on this information are, in the short term, giving results equivalent to those following more radical techniques (Forrest *et al.*, 1974).

We therefore decided that two controlled, randomized trials should be mounted in Edinburgh where for many years the orthodox primary treatment for clinical stages I and II breast cancer has been simple mastectomy and post-operative radical radiotherapy.

These trials are designed to answer two questions:

1. If histological examination of those nodes which are available at the time of simple mastectomy without dissecting the axilla (nodes of the pectoral or 'axillary-tail' group) shows no evidence of invasion by tumour, does immediate post-operative radical radiotherapy confer any advantage over a watching policy?
2. If the histological examination of the nodes reveals metastatic disease, does the addition of systemic therapy to conventional local treatment improve survival rates (Fisher *et al.*, 1975)?

As the objective of the second trial is to determine the effect of additional systemic therapy in disease of likely incurability, patients with clinically locally advanced (stage III) disease have been included. For them the conventional local treatment is either simple mastectomy and post-operative radiotherapy or radiotherapy alone.

### PATIENTS AND METHODS

The trials were designed by a steering committee composed mainly of representatives of general surgeons and radiotherapists in the South East Region of Scotland. Protocols were drawn up and circulated. Thirty-one surgeons and 6 radiotherapists agreed to take past. Both trials commenced, with MRC support, on 1 April 1975. They can be summarized as follows:

### *Trial I*

*Admission.*—Patients of TNM clinical stages I and II in whom histological examination of a pectoral node taken at simple mastectomy showed no evidence of metastatic tumour or in whom no node could be found for examination.

*Exclusions.*—Patients over 70 years of age, with previous malignant disease, with previous bilateral oophorectomy or hysterectomy or in poor general condition.

*Stratification.*—According to clinical size of tumour, site of tumour and menstrual status.

*Treatment.*—Randomization within 12 subgroups for radical post-operative radiotherapy (4250–4500 rads to max. in 10 fractions over 4 weeks) or for a watching policy (no immediate post-operative radiotherapy). In the event of previously palpable nodes enlarging or new nodes appearing in the watched group of patients, these would not be taken to mean failure of treatment. Histological confirmation of nodal involvement would lead to the patients being allocated as in trial II to receive either radical radiotherapy or radical radiotherapy plus chemotherapy using 5-fluorouracil.

*Assessment.*—Morbidity, disease-free interval, survival.

### *Trial II*

*Admission.*—(i) Patients with tumours of TNM clinical stages I and II who, following histological confirmation of involvement of a pectoral node, have received radical radiotherapy following simple mastectomy. (ii) Patients with tumours of TNM clinical stage III who have been treated either by radical radiotherapy alone or by simple mastectomy and radical radiotherapy according to the operability of the lesion.

*Exclusions.*—Patients over 70 years of age, with previous malignancy, with previous oophorectomy or hysterectomy, or who are considered unfit for chemotherapy.

*Stratification.*—According to the stage (stage II histological, stage III operated, stage III not operated) and the menstrual status.

*Treatment.*—Randomization within 9 subgroups for additional systemic therapy or inclusion in a matched control group when no additional therapy is given. Initially, the form of additional systemic therapy is chemotherapy, 5-fluorouracil, 700 mg/m$^2$ i.v. every 4 weeks for 12 injections.

### COMMENT

Within these trials, all patients with cancer of the breast of clinical stages I and II and stage III (operable) are treated by one standard operation, namely, simple (total) mastectomy. In the case of those with clinical stages I and II disease, the decision for subsequent additional treatment depends upon the histological findings in those nodes (pectoral) which are either removed with the axillary tail of the breast or identified during its dissection. While planned around the orthodox Edinburgh treatment of simple mastectomy and immediate post-operative radical radiotherapy, the design of these trials could be applied to other standard treatment policies, *e.g.*, *en bloc* resection of the breast and axillary nodes.

In these trials we are seeking to reduce the extent of local therapy, and therefore morbidity, for disease which, by histological assessment of nodal invasion, is shown to be confined to the breast. Local treatment is not enough when proof of local advancement is obtained either on clinical examination (stage III) or by examination of the removed pectoral nodes (histological stage II). In these circumstances improved survival rates are likely to be achieved only by the addition of systemic therapy to conventional treatment.

We are grateful for the help of Dr Mary Douglas, Mr A. J. Duff, Mr G. Meikle, Dr M. M. Roberts and Professor Sir Michael Woodruff who were members of the initial steering committee.

### REFERENCES

BRINKLEY, D. & HAYBITTLE, J. L. (1966) Treatment of Stage II Carcinoma of the Female Breast. *Lancet*, ii, 291.

BRINKLEY, D. & HAYBITTLE, J. L. (1971) Treatment of Stage II Carcinoma of the Female Breast—letter to Editor. *Lancet*, ii, 1086.

BRINKLEY, D. & HAYBITTLE, J. L. (1975) The Curability of Breast Cancer. *Lancet*, ii, 95.

CANT, E., SHIVAS, A. A. & FORREST, A. P. M. (1975) Lymph Node Biopsy during Simple Mastectomy. *Lancet*, i, 995.

COLE, M. P. (1964) The Place of Radiotherapy in the Management of Early Breast Cancer: A Report of Two Clinical Trials. *Br. J. Surg.*, **51**, 216.

EASSON, E. C. (1968*a*) Post-operative Radiotherapy in Breast Cancer. In *Prognostic Factors in Breast Cancer.* Ed. A. P. M. Forrest and P. B. Kunkler. Edinburgh: E. & E. S. Livingstone Ltd. p. 118.

EASSON, E. C. (1968*b*) In *The Curability of Cancer at Various Sites—The Breast.* London: Pitman Medical Publications. I.C.D. No. **170**, p. 59.

FISHER, B., SLACK, N. H., CAVANAUGH, P. J., GARDENER, B. & RAVDIN, R. G. (1970) Postoperative Radiotherapy in the Treatment of Breast Cancer: Results of the NSABP Clinical Trial. *Ann. Surg.*, **172**, 711.

FISHER, B., CARBONE, P., ECONOMOU, S. G., FRELICH, R., GLASS, A., LERNER, H., REDMOND, C., ZELEN, M., BAND, P., KATRYCK, L., WOLMARK, N. & FISHER, E. R. (1975) L-Phenylalanine Mustard in the Management of Primary Breast Cancer. *New Engl. J. Med.*, **292**, 117.

FORREST, A. P. M., ROBERTS, M. M., PREECE, P., HENK, J. M., CAMPBELL, H., HUGHES, L. E., DESAI, S. & HULBERT, M. (1974) The Cardiff–St Mary's Trial. *Br. J. Surg.*, **61**, 766.

HAMILTON, T., LANGLANDS, A. O. & PRESCOTT, R. J. (1974) The Treatment of Operable Cancer of the Breast: a Clinical Trial in the South-East Region of Scotland. *Br. J. Surg.*, **61**, 758.

KAAE, S. & JOHANSEN, H. (1968) Simple *versus* Radical Mastectomy in Primary Breast Cancer. In *Prognostic Factors in Breast Cancer.* Ed. A. P. M. Forrest and P. B. Kunkler. Edinburgh: E. & E. S. Livingstone Ltd. p. 93.

MURRAY, G. J. (1974) Cancer Research Campaign Breast Study. *Br. J. Surg.*, **61**, 722.

ROBERTS, M. M., FORREST, A. P. M., BLUMGART, L. H., CAMPBELL, H., DAVIES, M., GLEAVE, E. N., HENK, J. M., KUNKLER, P. B., SHIELDS, R., HULBERT, M., JAMIESON, C. W. & SELLWOOD, R. A. (1973) Simple *versus* Radical Mastectomy. Preliminary Report of the Cardiff Breast Trial. *Lancet*, i, 1073.

SUTHERLAND, I. (1974) In Proceedings of MRC Conference on Breast Cancer, July 1974.